# Pharmacogenetic Guided Opioid Therapy Improves Chronic Pain Outcomes and Comorbid Mental Health: A Randomized, Double-Blind, Controlled Study

**DOI:** 10.3390/ijms241310754

**Published:** 2023-06-28

**Authors:** Laura Agulló, Isidro Aguado, Javier Muriel, César Margarit, Alba Gómez, Mónica Escorial, Astrid Sánchez, Alicia Fernández, Ana M. Peiró

**Affiliations:** 1 Pharmacogenetic Unit, Clinical Pharmacology Department, Alicante Institute for Health and Biomedical Research (ISABIAL), General University Hospital of Alicante, c/Pintor Baeza, 12, 03010 Alicante, Spain; 2 Clinical Pharmacology, Toxicology and Chemical Safety Unit, Institute of Bioengineering, Miguel Hernández University, Avda. de la Universidad s/n, 03202 Elche, Spain; 3 Pain Unit, Department of Health of Alicante, General University Hospital of Alicante, c/Pintor Baeza, 12, 03010 Alicante, Spain; 4 San Vicente del Raspeig II Health Center, c/Alicante, 78, Sant Vicent del Raspeig, 03690 Alicante, Spain

**Keywords:** pharmacogenetics, *CYP2D6*, *OPRM1*, *COMT*, opioids, chronic pain

## Abstract

Interindividual variability in analgesic response is at least partly due to well-characterized polymorphisms that are associated with opioid dosing and adverse outcomes. The Clinical Pharmacogenetics Implementation Consortium (CPIC) has put forward recommendations for the *CYP2D6* phenotype, but the list of studied drug-gene pairs continues to grow. This clinical trial randomized chronic pain patients (*n* = 60), referred from primary care to pain unit care into two opioid prescribing arms, one guided by *CYP2D6*, μ-opioid receptor (*OPRM1*), and catechol-O-methyl transferase (*COMT*) genotypes vs. one with clinical routine. The genotype-guided treatment reduced pain intensity (76 vs. 59 mm, *p* < 0.01) by improving pain relief (28 vs. 48 mm, *p* < 0.05), increased quality of life (43 vs. 56 mm *p* < 0.001), and lowered the incidence of clinically relevant adverse events (3 [1–5] vs. 1 [0–2], *p* < 0.01) and 42% opioid dose (35 [22–61] vs. 60 [40–80] mg/day, *p* < 0.05) as opposed to usual prescribing arm. The final health utility score was significantly higher (0.71 [0.58–0.82] vs. 0.51 [0.13–0.67] controls, *p* < 0.05) by improving sleepiness and depression comorbidity, with a significant reduction of 30–34% for headache, dry mouth, nervousness, and constipation. A large-scale implementation analysis could help clinical translation, together with a pharmaco-economic evaluation.

## 1. Introduction

Treatment of moderate to severe nociceptive chronic pain requires a multidisciplinary approach, in which opioid analgesics continue to be standards of care. Given the chronic pain and mental health overlap, growing concerns about the opioid epidemic, and lack of data that support the use of opioids for long-term pain management, new treatment approaches are needed [1]. In the last few decades, genetic polymorphisms have proven to be one of the main culprits of analgesic response variability [2].

Pharmacogenomics (PGx) is the science of the relation between a person’s genetic attributes and drug response, which has been proposed as a tool to enhance opioid selection and optimization, with recent studies demonstrating successful implementation and outcomes [3]. The first guidelines related to opioid prescribing from the Clinical Pharmacogenetics Implementation Consortium (CPIC) were released almost 10 years ago [4]. These guidelines, which were updated in 2021, reviewed the evidence for *CYP2D6*, *OPRM1,* and *COMT* [5]. All three genes have biologically plausible mechanisms for affecting opioid response (*CYP2D6*—drug metabolism, *OPRM1*—drug target, *COMT*—pain perception) [5].

The *CYP2D6* gene is highly polymorphic, which translates to enzymatic activities ranging from non-functional (i.e., poor metabolizers; PMs) to increased function (i.e., ultrarapid metabolizers; UMs). The CYP2D6*3, *4, *5, and *6 alleles are non-functional alleles that occur secondary to frameshift mutations (*3, *6), splicing defects (*4) or whole gene deletion (*5) [6]. The frequencies of the PMs and UMs phenotypes range from 5 to 10% and 5 to 11%, respectively, in different populations [7]. In fact, PMs carry two non-functional alleles that give no activity to the enzyme; this would mean decreased levels of the active metabolite, leading to a lack of analgesic effectiveness [8]. Compared to normal metabolizers (NMs), PMs have lower concentrations of the active metabolites of codeine, tramadol, hydrocodone, or oxycodone [9,10,11]. Intermediate metabolizers (IMs) possess one non-functional and one reduced-function allele and have significantly reduced enzyme activity compared to NMs. Albeit less studied, IMs are at risk of the reduced biotransformation of *CYP2D6*-mediated opioids into more potent metabolites by conferring decreased analgesia [12,13]. Finally, UMs have several copies of the gene, and the activity is higher than normal, so they would present increased levels of the most active molecule, which means that the metabolism of various opioids could be more efficient in these individuals, with lower analgesic doses required [14]. However, UMs would be more susceptible to opioid toxicity, including the development of adverse events [15].

The μ-opioid receptor is the main molecular target for analgesia. The *OPRM1* gene, which codes for this receptor, is located at chromosome 6q24-q25, consisting of 4 exons and 3 introns [16]. More than 250 SNPs have been described for this gene; they have always been primary candidates for demonstrating genetic influence on the analgesic response to opioids. The most studied SNP in the field of pain is A118G (rs1799971), which consists of the change of adenine to guanine at position 118, leading to the loss of an N-glycosylation site in the extracellular region [17]. At a molecular scale, it has been observed that the presence of the polymorphism causes a greater affinity for β-endorphin compared to the wild-type variant [13], leading to a decrease in the perception of pain intensity and a lower cortical response to the painful stimulus in G homozygotes. Moreover, the prevalence of this polymorphism is estimated to be between 10 and 19%, and the distribution of alleles may be different depending on ethnicity [18].

The enzyme Catechol-O-Methyltransferase, encoded by the *COMT* gene, is involved in the degradation process of catecholamines, a group of neurotransmitters such as adrenaline, noradrenaline, or dopamine. The G472A variant (rs4680), one of its most studied polymorphisms, results in a substitution of a valine residue for methionine at codon 158 (Val158Met) [19]. In human studies, this variant has been associated with a decreased response of the opioid system and a higher density of μ-receptors, which may lead to increased effectiveness, especially related to morphine and fentanyl [20,21]. Furthermore, as serotonin interacts with dopamine and dopamine availability is influenced by *COMT* SNP [22], interindividual differences in response to antidepressants response and other psychiatric phenotypes such as schizophrenia have been evidenced [23,24].

Although there is the potential for improved pain management through PGx, as well as several practice-based examples of PGx-guided opioid optimization, these practices have yet to become mainstream [25] in the pain and mental health comorbidity management context. The aim of the present study is to evaluate the effectiveness and safety of PGx-guided opioid therapy by analyzing clinical changes in patients with chronic non-cancer pain (CNCP) after 3 months of opioid treatment. The PGx-guided treatment used was based on the CPIC clinical recommendations for *CYP2D6* phenotypes, *OPRM1*, and *COMT* genotypes.

## 2. Results

### 2.1. Patient Demographic and Genetic Data

Sixty Caucasian subjects, all living in Spain, were included. Most were naïve to opioids and referred from primary care to pain unit care for opioid prescribing given CNCP intensity. During the 3-month trial, 10/60 (17%) patients were excluded (*n* = 2 PGx-guided arm, *n* = 8 usual care) due to no opioid adherence (*n* = 7), with one missing case (*n* = 1) and not enough saliva samples (*n* = 2). In the end, 50 adult patients (80% women vs. 20% men, with a significantly higher percentage of females in the PGx-guided arm), whose mean age was 59 ± 14 years old, were predominantly married with inadequate financial resources (22% on disabled government pay), completed the 3-month follow-up. The genotype distribution was balanced between both arms. Mutant genotypes *OPRM1*-GG (8%), *COMT*-AA (36%), and the extreme phenotypes of the *CYP2D6* gene, PMs and UMs, appeared as 4–6%. A summary of the differences in the demographic and genetic data results between the PGx-guided and usual care arms appears in Table 1.

### 2.2. Pain Management Efficacy

**Figure 1:**
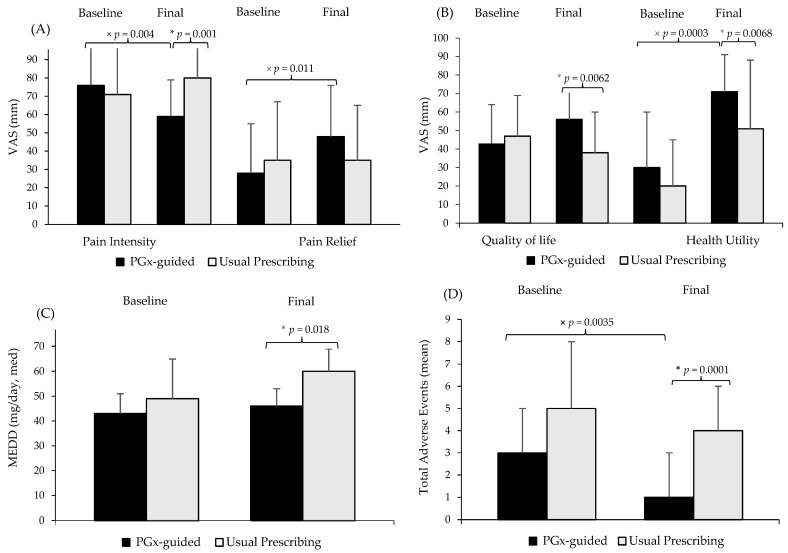
Analysis of the clinical and pharmacology data at the baseline and after the 3-month opioid titration procedure (final visit) by comparing the pharmacogenetic-guided therapy (cases) to usual prescribing (controls). * Denotes *p* < 0.05, comparing cases vs. controls. × Denotes *p* < 0.05 comparing baseline vs. final visit. (**A**) pain intensity and pain relief (visual analog scale (VAS), 0–100 mm); (**B**) quality of life (VAS, 0–100 mm) and health utility (0–100) (**C**) MEDD, morphine equivalent daily dose (mg/day, median); (**D**) total adverse events (mean).

Figure 1 illustrates the significant differences derived from analyzing the clinical (A, B) and pharmacological (C) data of the PGx-guided arm vs. usual care (baseline and 3 months after opioid treatment). At the baseline visit, the clinical outcomes between both arms did not show any significant differences. In the final visit, the PGx-guided arm showed a significant reduction in perceived pain intensity (76 vs. 59 mm, *p* < 0.01) with increased relief (28 vs. 48 mm, *p* < 0.05). In relation to the 3-month quality of life outcomes, health utility significantly increased in the PGx-guided arm (median [IQR] baseline vs. final visit, 0.30 [0.06–0.65] vs. 0.71 [0.58–0.82], *p* < 0.05). In the usual care arm, final pain intensity was significantly higher (80 vs. 59 mm, *p* < 0.01), with worse quality of life (38 vs. 56 mm, *p* < 0.05) and health utility (0.51 [0.13–0.67] vs. 0.71 *p* < 0.05) [0.58–0.82]) compared to the PGx-guided arm, and required 42% higher morphine equivalent daily dose (MEDD) (median [IQR], control vs. cases, 60 [40–80] vs. 35 [22–61] mg/day, *p* < 0.05).

Table 2 reports all the clinical and pharmacological variables. Upon the baseline visit, the PGx-guided arm received a significantly higher 68% for simple analgesics. Conversely, the usual care arm showed 18% higher fentanyl use at the final visit (*p* < 0.05). For mental health, the PGx-guided arm showed a significant reduction in anxiety values (HAD, median of 8 [5–11] vs. 5 [3–7] scores, *p* < 0.01), and an improvement in the Short Format Health Survey 12 (SF12) results (27 vs. 36 scores, *p* < 0.01), which evaluates the physical component of health, meanwhile any significant difference was observed in usual care arm.

### 2.3. Pain Management Tolerability

A reduction was observed at 3 months in the total number of adverse events (AEs) (3 [1–5] vs. 1 [0–2], *p* < 0.01) in the PGx-guided arm vs. the baseline visit, as seen in Table 3 and Figure 1D, with no identified harm or adverse drug reactions related to the intervention. An incidence rate of 3–4 AEs/patient was obtained. At the baseline visit, the most frequent disorders (>20%) were psychiatric (nervousness, depression), nervous (headache, sleepiness, insomnia, dizziness), and gastrointestinal (constipation, dry mouth, weight change). At the final visit, the PGx-guided arm showed a lower AEs/patient rate (1 [0–2] vs. 4 [2–6], *p* < 0.001), with less than 23% prevalence for sleepiness (cases vs. controls, OR (95%CI) = 0.06 [0.003–1.07]); 30% prevalence for nervousness (cases vs. control., OR (95%CI) = 0.17 [0.040–0.75]); 34% prevalence for headache (cases vs. controls, OR (95%CI) = 0.15 [0.030–0.62]), and 27% prevalence for constipation (cases vs. controls, OR (95%CI) = 0.24 [0.062–0.93]) as opposed to the usual care arm. A 32% lower prevalence of dry mouth was also observed (cases vs. controls, OR (95%CI) = 0.22 [0.060–0.78]), although this significant difference also appeared at the baseline visit (55 vs. 50%, *p* < 0.05). Insomnia (usual care arm, 59 vs. 23%, *p* < 0.05), sleepiness (PGx-guided arm, 25 vs. 0%, *p* < 0.05), and depression (PGx-guided arm, 25 vs. 0%, *p* < 0.05) improved upon the final visit.

## 3. Discussion

The clinical decision about whether to use opioids for CNCP should be guided by a risk-benefit consideration. This trial provides data on the clinical efficacy of the *CYP2D6* phenotype plus *OPRM1*/*COMT* genotype and guided opioid selection for improving pain management improving quality of life and physical health, and for reducing opioid dose-related risks, including and comorbid mental health symptoms, compared to usual prescribing. A significant and positive effect on pain relief, anxiety levels, and health utility status was found at the 3-month follow-up only in the PGx-guided arm. Pharmacogenomic testing provided relevant clinical information during the opioid titration decision process in randomized CNCP patients vs. usual prescribing. Ultimately, future clinical implementation would need a cost-utility analysis, in larger samples, from the health system perspective.

The advantages of using pharmacogenetics-based opioid therapy before analgesic prescription are well-documented. The present results showed that the group treated with genetic information indicated a significantly less pain intensity and improved physical function compared to the conventionally treated group. However, very few prospective studies have assessed clinical utility. A large retrospective economic analysis developed in 5288 patients genotyped adult patients in front of 22,357 controls has reported reduced direct medical charges per patient of around US$7000 [26]. These data support the clinical implementation of pharmacogenetic-panel testing in real-world scenarios [27].

Moreover, the influence of pharmacogenetics on analgesic pharmacokinetics can be a determined factor in understanding interindividual analgesic response. In particular, 27% of the European population struggles with the proper metabolism of *CYP2D6* drugs that can modify tramadol and oxycodone metabolites based on O-demethylation [28] (Table S1). This could avoid cardiotoxicity and respiratory depression in UMs patients under tramadol prescriptions [29,30] together with emergency department visits in opioid longer users [31]. Interestingly, this can have a sex impact due to the higher tramadol female prescriptions in front of men recorded in national databases from Denmark, Norway, and Sweden [32]. This tendency has been confirmed in the Spanish population, where females have a higher number of tramadol prescriptions compared to a higher dose for males [33]. These differences should be considered with drug abuse cases or even as a predictor of failed analgesic therapies, which should include a sex-differences analysis.

On the other hand, psychiatric needs in chronic pain patients remain challenging, even more in the south of Europe, where the number of public resources in mental health is lower [34,35]. In previous studies, depression, anxiety, sleep disturbance, and finally, physical disability significantly impacts chronic pain patients, according to the literature [36]. Here, a special warning must be given related to the impact of the waiting list in chronic pain patients remitted to pain units [37] and to the high antidepressive, anxiolytics antidepressants (mostly tricyclic and serotonin selective reuptake inhibitors), and neuromodulators drug co-prescribed in our sample. The safety impact requires special caution when these drugs are used with opioid analgesics [38]. It is important to be aware that some of them act as inhibitors of the *CYP2D6* enzyme. Otherwise, when benzodiazepines and anticonvulsants are opioids co-prescribed, they could impact opioid levels. More attention should be paid because genotype could avoid some of the side effects registered [39]. Thus, psychiatric and sleep comorbidities management should be incorporated into routine CNCP patient care [36], even more in primary health [40].

Despite the promising results, it is crucial to acknowledge that current research into using genetic information to guide pain treatment is still in its early stages. Baseline screening of any prescribed drug side effects is needed through validated scales before starting any new regimen for pain care. This includes routinely screening and monitoring patient perceptions and avoiding drug-drug interactions with PGx-guided decisions. Future algorithms to interpret the *CYP2D6* phenotypes and machine learning knowledge are needed, with an inclusive representation of understudied populations with special attention to sex or ethnic background differences [41].

Therefore, additional studies are necessary to confirm and extend these findings by considering the cost of genetic testing, which may pose challenges when implemented into routine clinical practice. Nonetheless, the potential benefits of this novel approach have significant implications for improving pain management and enhancing patients’ overall well-being.

### Limitations

This study has some constraints that need to be noted. First, the sample size was limited by a “convenience sample” from a single pain unit center during the COVID-19 pandemic period. This could have affected the representativeness of the population and prevented us from detecting statistically significant differences related to gene variants or from an additional sex-differences analysis. The results presented in this paper must be taken cautiously, as it would be necessary to increase the final number of patients in the study. In this regard, some post hoc analysis may be helpful to replicate the analyses with a larger number of patients in the future. Furthermore, as patients were on concomitant medication to treat other pathologies, unmeasured factors could have contributed to the observed differences, especially in relation to drugs’ AEs. They could have independently contributed to the observed AEs and differences in pain care [42,43]. It should be noted that the prescription of specific opioids was adapted to each patient’s specific needs. Although we obtained a balanced distribution of drugs between the two comparison groups, it should be mentioned that this was not the case for the opioid fentanyl, which was only used in the control arm, and it is difficult to evaluate these specific effects of the guided treatment if there is no balance of drugs between the groups.

It should also be noted that there is a significant pharmacological pain management and psychological disorders overlap. The patients with psychiatric diagnoses (i.e., major depression, generalized anxiety, obsessive-compulsive or panic disorders), and those who used illicit drugs or medical cannabinoids, were excluded from participating in the study. Other important factors were not controlled in this study, such as female predominance, pain duration, or variables such as body mass index (MEDD was not adjusted according to body weight), testosterone/estrogen levels, or other lifestyle influences. They could have all interfered with the occurrence of pain experience and analgesic response. In this situation, they could have independently influenced the recorded side effects and should be considered in future studies.

## 4. Materials and Methods

### 4.1. Study Design and Participants

**Figure 2:**
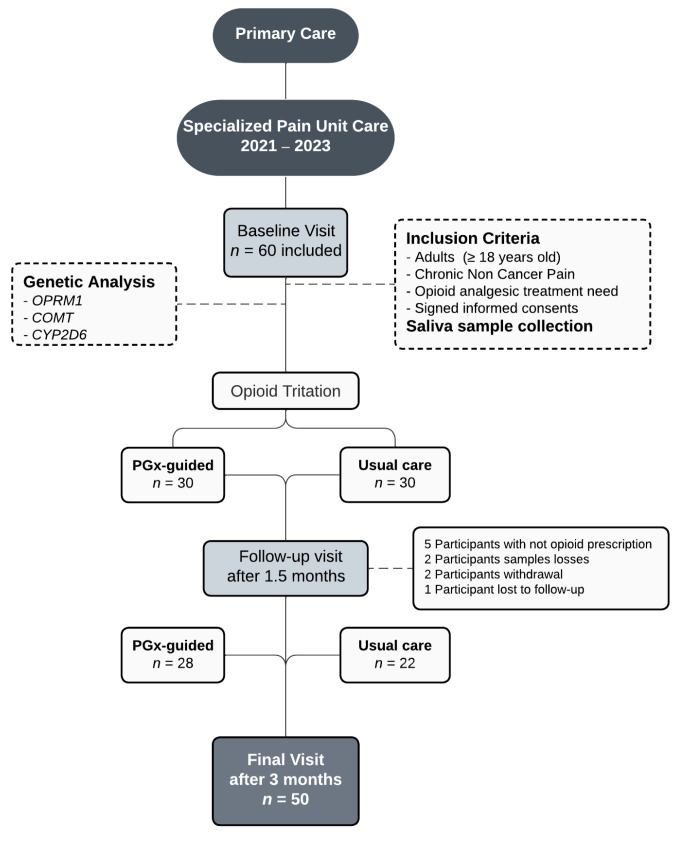
Study flow chart from the baseline to the final visit after 3 months of opioid titration.

A clinical trial was designed and developed at the pain unit (PU) of the Alicante Health Department of the Dr. Balmis General University Hospital, Spain, from October 2021 to April 2023, with patients referred to from six primary care centers. Sixty patients with CNCP (47 women and 13 men) were included. The inclusion criteria were adults aged ≥ 18 years with CNCP who required opioid analgesic treatment and signed informed consent. Patients under 18 years with oncologic pain or any psychiatric disorders that could interfere with properly conducting this study were excluded. This study did not incorporate other chronic pain conditions of unknown pathophysiologies, such as fibromyalgia or neuropathic pain conditions, such as painful polyneuropathy, postherpetic neuralgia, trigeminal neuralgia, and post-stroke pain. The study flow chart is shown in Figure 2.

**Figure 3:**
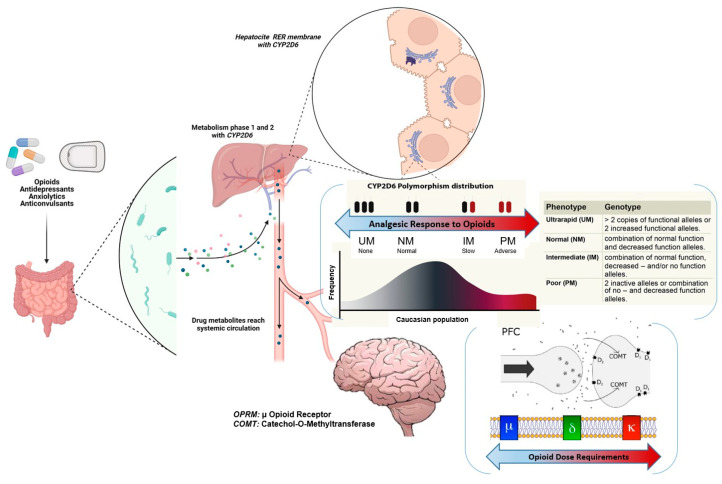
Description of the relation between the different opioids and coadjutant analgesic drugs with the *CYP2D6*, *OPRM1,* and *COMT* genes. Adapted from Ballester et al., 2022. [39] PFC: prefrontal cortex, RER: rough endoplasmic reticulum.

A consecutive sampling method was used in ambulatory patients. The researchers reviewed the schedule of all the weekly pain unit patients, one day a week, usually on Monday, for prescreening, and prepared the questionnaires and informed consent. When a patient met the inclusion criteria, he/she was informed about the purpose of the study by the pain unit healthcare team. Then the interested individuals were attended to by the research staff to sign the informed consent and collect all the variables. If necessary, they were completed using Electronic Health Records (EHRs), which allow medical diagnoses, outcomes, and medication use to be reviewed. When participants enrolled in this study, saliva samples were collected for the pharmacogenetic analysis. Approximately 2 mL of saliva was collected in tubes containing 5 mL of PBS. These tubes were rigorously identified and sent to the laboratory for next-day genotyping. Here the researchers randomly allocated patients to one of the two arms using the clinical trial management software OxMaR (v2014). They reported only the genotype of the patients in the PGx-guided arm to clinicians and indicated any variants that could influence opioid management. The *CYP2D6*, *OPRM1,* and *COMT* genes were used to guide opioid treatment (Figure 3). This information was registered in the EHRs to be accessed by any health medical staff member. The follow-up period compromised a minimum of one first visit (around the first month) and the final visit at 3 months.

### 4.2. Data Collection and Opioid Titration

All the patients were interviewed upon the baseline visit to assess their physical health and medical history. Validated scales and questionnaires were completed during the baseline and final visits (after 3 months of opioid treatment). All the questionnaires were self-administered but supported by the presence of an expert clinician.

#### 4.2.1. Clinical Outcomes

A Global Pain State questionnaire, which qualitatively measures pain intensity and relief, was determined on the validated 100-mm visual analog scale (VAS; 0 = “no pain/relief” to 100 = “worst possible pain/maximum relief”) [44]. Quality of life related to health measures was assessed by the EuroQol VAS (EQ-VAS; 0 = “worst” to 100 = “best health status”). Health utility status (0 death, 1 perfect health) was measured with the dimensions of mobility, self-care, usual activities, pain/discomfort, and anxiety/depression (reg. no. 53112, available at https://euroqol.org/, accessed on 15 June 2023). The Short Format Health Survey 12 (SF12), a 12-item questionnaire with a mental (MCS, 19–61 scores) and a physical component score (PCS, 24–57 scores), and a mean of 50 and a standard deviation of 10 in the general US population [45], was also used. Psychological status was calculated according to Hospital Anxiety and Depression Scale (HADS, 0–21 scores, classified as normal (<7), probable (8–10), and case (>11 scores)) [46].

#### 4.2.2. Pharmacology and Safety Data

After inclusion, upon the baseline visit, the required opioid prescription was initiated according to diagnosis for a 3-month treatment period according to the patient’s degree of compliance and preferences (oral or transdermal). The prescription was made by four anesthesiologists and re-evaluated by two research team members.

Physicians recorded patients’ prescribed pain therapy (opioids, i.e., tramadol, fentanyl, tapentadol, buprenorphine, oxycodone, morphine, methadone) and concomitant drugs, neuromodulators (pregabalin and gabapentin), antidepressants (i.e., amitriptyline, fluoxetine, escitalopram, and duloxetine), anxiolytics, simple analgesics (i.e., paracetamol and metamizole), and non-steroidal anti-inflammatory drugs (NSAIDs). With different opioid combinations, the oral morphine equivalent daily dose (MEDD) was estimated using available references [47]. A questionnaire with a list of the most frequently selected AEs, according to the summary of the opioid product characteristics at a frequency of “very common” or “common”, plus a blank field to add any other AEs, was developed by our group to record all the AEs reported by patients.

### 4.3. Genetic Determination

After collecting the saliva sample, it was preserved at a temperature of −80 °C until further processing. The extraction of genomic DNA was performed using the E.N.Z.A. forensic DNA kit (Omega Bio-Tek Inc., Norcross, GA, USA), following the guidelines provided by the manufacturer. For this particular study, the samples were obtained from Alicante BioBank and processed following standard operating procedures.

The following genetic variants were genotyped at the Molecular Biology Laboratory of ISABIAL (Alicante, Spain), and their results were used to guide opioid treatment: *OPRM1* (A118G, rs1799971), *COMT* (G472A, rs4680) and CYP2D6*2 (1584C > G), *3 (2550delA), *4 (1847G > A), *5 (*CYP2D6* full gene deletion), *6 (1708delT), *10 (100C > T), *17 (1022C > T), *29 (3184G > A), *35 (31G > A), *41 (2989G > A) polymorphisms, using the real-time PCR rotor gene Q system (Qiagen, Hilden DE-NW, Germany) with specific TaqMan MGB^®^ probes (Applied Biosystems, Pleasanton, CA, USA). The amplification parameters were as follows: pre-PCR section, 10 min at 95 °C, 40 cycles for 15 s denaturation at 92 °C, and 1 min final extension at 60 °C. For the *CYP2D6* gene, a standard estimation of its metabolic phenotype, based on its enzymatic activity, null function (PMs), reduced function (IMs), normal function (extensive metabolizer, EM), and increased function (UMs) [5], was performed from its genotype. Each allele was assigned a consistent activity score with CPIC recommendations. The recommendations are based on the activity score (AS) scoring system [48]. To calculate it, alleles are grouped according to their functionality, and a value is assigned to each allele: non-functional alleles (*3, *4, *5, and *6, give a score equal to 0), alleles of decreased function (0. 25 for *10, and 0.5 for *17, *29 and *41), normal function alleles (*1, *2 and *35, have a value of 1) and increased function alleles (*1×N, *2×N and *35×N, give a score of 2). The phenotype was assigned based on the sum of scores (i.e., gene score): 0, PMs; 0.5, IMs; 1–2, EMs; and > 2, UMs. We analyzed the copy number of the *CYP2D6* gene following the TaqMan Copy Number Assays (Thermo Fisher Scientific, Waltham, MA, USA), which is run together with a TaqMan Copy Number Reference Assay (Thermo Fisher Scientific, Waltham, MA, USA) in a duplex qPCR reaction. In case of duplications, the possible activity scores were calculated for each of the alleles. In all cases where it was present (4), regardless of whether the allele was active or inactive, this did not affect the estimated metabolizer phenotype taken into account for the analysis. The genotype and phenotype outcomes were recorded as categorical variables within the electronic health records (EHRs) under the laboratory results section, aiming to provide electronic clinical decision support. This system alerts healthcare providers regarding the PMs, IMs, or UMs phenotype if there is a future prescription of a *CYP2D6*-metabolized opioid.

### 4.4. Recommendations Based on Pharmacogenetics

For the participants in the PGx-guided arm, physicians had opioid prescribing recommendations based on the assigned *CYP2D6* phenotype and the *OPRM1* and *COMT* genotypes (Table 4). Similar to the CPIC guidelines, recommendations focused on *CYP2D6*-metabolized opioids (codeine, tramadol, oxycodone, hydrocodone), with a recommended alternative treatment in PMs and UMs due to the inefficacy and toxicity risk, respectively. A key difference between the CPIC guidelines and the recommendations provided in this trial lies in the alternative therapy also being recommended for IMs taking codeine, tramadol, oxycodone, and hydrocodone if the pain was not well-controlled. In addition, *CYP2D6* drug interactions were considered, as the presence of *CYP2D6* inhibitors may decrease or increase the efficacy of certain drugs (Table S2). The recommendation was delivered to the physician via a clinical consultation note in the EHRs within one week after the baseline visit. The genotyping results were not actively communicated to patients as part of the study.

### 4.5. Statistical Data Analysis

The Shapiro–Wilks normality test was performed to choose a parametric or non-parametric test for comparisons. Quantitative data are presented as the mean standard deviation (SD) or the median interquartile range [IQR]. Categorical variables are expressed as percentages. The relative frequencies of genotypes were calculated for each group. The observed gene frequencies were compared with expected frequencies using the chi-square test and the Hardy–Weinberg proportion. In all cases, multiple testing was adjusted by the Bonferroni correction method. The demographic, clinical, pharmacological, and genetic data were compared using either the χ^2^ or Fisher’s exact test for the categorical variables and the t-test or Mann–Whitney U test for the continuous variables depending on their distribution. When more than two groups were involved, ANOVA/Kruskal–Wallis or chi-square tests were used for the continuous or categorical variables. Statistical analyses were performed with the R software (v 4.0.3, Auckland, CA, USA) and GraphPad Prism, version 5.02 (GraphPad Software, La Jolla, CA, USA). *p* < 0.05 was considered to be statistically significant. We analyzed the statistical power of our results with a sample size of 28 cases and 22 controls using the statistical program Epidat 4.2. (Sergas, A Coruña) with a confidence level of 95%, and we obtained a statistical power of 93%, expecting to detect at least a 30% difference between the means [49].

## 5. Conclusions

Our results support the efficacy and safety of the clinical implementation of genotype-guided CNCP opioid use for pain and comorbid psychiatric disorders management, lowering the risk of iatrogenically induced side effects and opioid dose requirements compared to usual care. Caution and special consideration are needed before administering anxiolytics, particularly benzodiazepines, antidepressants, or neuromodulators, to patients with chronic opioid use due to the high-risk use behavior in general adult populations.

## Figures and Tables

**Table 1:** Baseline demographic and genetic characteristics of chronic non-cancer pain (CNCP) patients in both clinical trial arms: pharmacogenetic (PGx) guided opioid therapy (cases) vs. usual routine care (controls).

CNCP Patients		Total (*n* = 50)	PGx-Guided (*n* = 28)	Usual Care (*n* = 22)
Women % (*n*)		80 (40)	93 (26) *	64 (14)
Age (years old, mean (SD))		59 (14)	59 (14)	60 (14)
Employment status % (*n*)				
Active		32 (16)	32 (9)	32 (7)
Disability		22 (11)	21 (6)	23 (5)
Retired		20 (10)	21 (6)	18 (4)
Homemaker		18 (9)	14 (4)	23 (5)
Unemployment		8 (4)	11 (3)	5 (1)
Genetic data % (*n*)				
*CYP2D6*	PMs	4 (2)	4 (1)	5 (1)
	IMs	42 (21)	43 (12)	41 (9)
	EMs	48 (24)	46 (13)	50 (11)
	UMs	6 (3)	7 (2)	5 (1)
*OPRM1*	AA	62 (31)	68 (19)	55 (12)
	AG	30 (15)	28 (8)	32 (7)
	Mutant-GG	8 (4)	4 (1)	14 (3)
*COMT*	GG	12 (6)	14 (4)	9 (2)
	GA	52 (26)	39 (11)	68 (15)
	Mutant-AA	36 (18)	46 (15)	23 (5)

* Denotes *p* < 0.05 comparing cases vs. controls (highest value shown in bold). SD: standard deviation; PMs: poor metabolizers, IMs: intermediate metabolizers, EMs: extensive metabolizers, and UMs: ultrarapid metabolizers.

**Table 2:** Clinical and pharmacological data of the chronic non-cancer pain patients in both clinical trial arms: pharmacogenetic (PGx)-guided opioid therapy (cases) vs. usual routine care (controls) before and after opioid treatment.

	Baseline		Final (3 Months)	
Mean (SD), median [IQR] or %	**PGx-guided** ***n* = 28**	**Usual care** ***n* = 22**	**PGx-guided** ***n* = 28**	**Usual care** ***n* = 22**
Pain intensity (0–100 mm)	**76 (21)** **××**	71 (26)	59 (20)	80 (19) **
Pain relief (0–100 mm)	28 (27)	35 (32)	**48 (29) ×**	35 (31)
Quality of life (0–100 mm)	43 (21)	47 (22)	56 (15) **	38 (22)
Health Utility (0–1 scores)	0.30 [0.06–0.65]	0.20 [0.03–0.65]	0.71 [0.58–0.82] * ×	0.51 [0.13–0.67]
HAD-Anxiety (0–21 sco.)	**8 [5–11] ×**	7 [5–10]	5 [3–7]	9 [5–12]
HAD-Depression (0–21 sco.)	7 [4–9]	8 [4–12]	4 [3–7]	8 [2–12]
Physical (SF12)	27 (7)	28 (7)	**36 (8) ××**	29 (9)
Mental (SF12)	43 (14)	41 (12)	43 (7)	43 (12)
MEDD (mg/day)	34 [23–56]	49 [36–112]	35 [22–61]	60 [40–80] *
Simple analgesics	68 *	36	71	64
NSAIDs	21	9	21	14
Tramadol	39	27	61	50
Fentanyl	0	9	0	18 *
Oxycodone	11	5	4	9
Tapentadol	0	5	**21** **×**	9
Buprenorphine	7	5	7	0
Neuromodulators	50	32	57	64
Antidepressants	36	41	39	50
Anxiolytics	43	45	32	45

HAD: Hospital Anxiety and Depression Scale; SF12: Short Format Health Survey 12; MEDD: morphine equivalent daily dose; NSAIDs: non-steroidal anti-inflammatory drugs. * Denotes *p* < 0.05, and ** denotes *p* < 0.01 comparing cases vs. controls. The highest value between cases and controls is shown in a gray background. × Denotes *p* < 0.05, and ×× denotes *p* < 0.01 comparing baseline vs. final visit (highest value shown in bold).

**Table 3:** Safety data of the chronic non-cancer pain patients in both clinical trial arms: pharmacogenetic (PGx)-guided opioid therapy (cases) vs. usual routine care (controls) before and after opioid treatment.

	Baseline		Final (3 Months)	
Median [IQR]) or %	**PGx-guided** ***n* = 28**	**Usual care** ***n* = 22**	**PGx-guided** ***n* = 28**	**Usual care** ***n* = 22**
Total Adverse Events	**3** **[1–5]** **××**	4 [3–6]	1 [0–2]	4 [2–6] **
Insomnia	29	59 * **×**	7	23
Sleepiness	**25** **×**	32	0	23 *
Depression	**25** **×**	18	0	5
Nervousness	29	45	11	41 *
Dry mouth	25	55 *	18	50 *
Headache	25	36	11	45 **
Constipation	21	32	14	41 *
Weight change	25	27	4	14
Dizziness	21	32	11	18
Itching	18	27	11	23
Loss of libido	11	14	7	5
Dry Skin	14	14	11	32
Nausea	7	23	7	5
Lack of appetite	7	18	7	18
Edema	4	5	0	5
Redness of skin	0	5	0	5
Sexual disturbance	0	9	0	9
Vomiting	0	14	0	5

* Denotes *p* < 0.05, and ** denotes *p* < 0.01 comparing cases vs. controls. The highest value between cases and controls is shown in a gray background. × Denotes *p* < 0.05, and ×× denotes *p* < 0.01 comparing the baseline vs. final visit. The highest value between the baseline vs. final visit is shown in bold.

**Table 4:** Recommendations for opioid prescribing based on the *CYP2D6* phenotype assigned and the *OPRM1* and *COMT* genotypes. Based on CPIC clinical guideline recommendations [5].

Opioids	Tramadol Codeine	Oxycodone Hydrocodone	Tapentadol Buprenorphine Fentanyl
*CYP2D6* Phenotye			
UMs	Avoid codeine and tramadol use because of serious toxicity potential	Use of oxycodone with caution/vigilance. Increase surveillance for increased adverse events	No recommendation
NMs	Use tramadol label recommended age-specific or weight-specific dosing	No recommendation	No recommendation
IMs	Use tramadol label recommended age-specific or weight-specific dosing	No recommendation	No recommendation
PMs	Avoid codeine and tramadol use because of the possible decrease in analgesic response	Use of oxycodone/hydrocodone with caution/vigilance	No recommendation
*OPRM1* Genotype			
AA	The presence of the AA wild-type genotype implies a lower dose required to achieve analgesia		
AG/GG	The presence of the G allele implies a higher dose required to achieve analgesia		
*COMT* Genotype			
AG/AA	The presence of the A allele implies a lower dose required to achieve analgesia		

## Data Availability

The data presented in this study are available on request from the corresponding author. The data are not publicly available because they contain clinical data of patients.

## Supplementary Materials

The supporting information can be downloaded at: https://www.mdpi.com/article/10.3390/ijms241310754/s1. Adapted from Ballester et al., 2022 [50,51,52,53,54,55,56,57,58,59,60,61,62,63,64,65,66].
