# Supplementary material for: Pharmacogenetic Guided Opioid Therapy Improves Chronic Pain Outcomes and Comorbid Mental Health: A Randomized, Double-Blind, Controlled Study
Source: Int J Mol Sci. 2023 Jun 28;24(13):10754. doi: 10.3390/ijms241310754 (PMC10341655; doi:10.3390/ijms241310754)
Supplement: Supplementary file 1 [file ijms-24-10754-s001.zip › ijms-2435097-supplementary.pdf]

SUPPLEMENTATY MATERIAL

Table S1: *CYP2D6* phenotype impact in opioid pharmacology outcomes. Adapted from Ballester et al., 2022 [39].

	Metabolism	Major findings	References
TRAMADOL	Dose adjustment	*CYP2D6* UMs and PMs guided prescribing improves analgesia, 24% of *CYP2D6*-dose guided vs. 0% non-guided reported ≥30% (clinically meaningful) reduction in the pain measurement	Smith et al., 2019 [50]
	Major doses	The consumption of tramadol in *CYP2D6**10/*10 group was significantly higher than that in *CYP2D6**1/*1 or *CYP2D6**1/*10 (P < 0.05)	Dong et al., 2015 [51]
	Decreased metabolism	*CYP2D6* UMs and PMs phenotypes are associated with a reduction of the O-desmethylation process compared to EMs	Bastami et al., 2014 [52]<br>Lane et al., 2014 [53]<br>Haage et al., 2018 [54]<br>Tanaka et al., 2018 [55]
	Decreased metabolism	*CYP2D6*4* or **10* have a decreased excretion of O-desmethyltramadol.	Arafa et al., 2018 [56]
	Decreased metabolism	Decreased rations of O-desmethyltramadol/tramadol ratio and N,O-desmethyltramadol/N-desmethyltramadol in *CYP2D6*5/*5* and **10/*10*	Yu et al., 2018 [57]<br>Yu et al., 2018 [58]
	Increased metabolism	N-desmethyltramadol/O-desmethyltramadol concentration ratios are increased in PM genotype	Fonseca et al., 2016 [59]
	Increased metabolism	*CYP2D6*10/*10* is associated with increased formation of N-desmethyltramadol from tramadol (p<0.05)	Yu et al., 2018 [57]
	Increased metabolism	*CYP2D6* UMs and PMs phenotypes are associated with an increase of the N-desmethylation process compared to EM	Haage et al., 2018 [54]<br>Tanaka et al., 2018 [55]
	Decreased and Increased metabolism	The plasma concentration of O-desmethyltramadol and its ratio to tramadol were lower in the *CYP2D6* IMs + PMs group than in NM group (P = 0.002 and P = 0.023). The plasma concentration of N-desmethyltramadol and its ratio to tramadol were higher in the *CYP2D6* IMs + PMs group than in the NMs group (P = 0.001 and P = 0.001)	Tanaka et al., 2018 [55]
	Equal metabolism	No statistical difference of tramadol concentrations between phenotypes	Bastami et al., 2014 [52]<br>Tanaka et al., 2018 [55]
	Increased side-effects	*CYP2D6* PMs increased frequency of tramadol-induced AEs	Elkalioubie et al., 2011 [30]<br>Orliaguet et al., 2015 [29]
	Major side-effects	In carriers of *9/*9, *5/*5, *5/*4, and *10/*10, also in 4 allele polymorphisms (*4/*1 [38.4%] and *4/*4 [42.8%])	Batiskayi et al., 2020 [60]
	Decreased side-effects	*CYP2D6*4* or **10* alleles are associated with decreased severity of tramadol-induced hepatotoxicity p<0.05	Arafa et al., 2018 [56]

**OXYCODONE**	Dose adjustment	*CYP2D6* UMs and PMs guided prescribing improves analgesia	Linares et al., 2014 [28]
	Major efficacy and side effects	*CYP2D6**6 and *9 carriers had a reduced (*9) or absent (*6) cytochrome activity, and therapeutic failure, and *CYP2D6* UMs showed an increased risk of side effects. Plus, *CYP2D6* *1/*11, *4/*6 and *41/* 2N showed higher efficacy and side effects when chronic opioid treatment	Dagostino et al., 2018 [61]
	Equal doses	*CYP2D6* PMs and EMs no statistical differences of oxycodone doses	Naito et al., 2011 [62]
	Reduced metabolism	*CYP2D6* PMs genotypes are associated with decreased exposure to oxymorphone and noroxymorphone	Stamer et al., 2013 [63]<br>Andreassen et al., 2012 [64]<br>Balyan et al., 2017 [65]
	Equal metabolism	No statistical differences of oxycodone concentrations	Naito et al., 2011 [62]<br>Andreassen et al., 2012 [64]<br>Balyan et al., 2017 [65]
	Equal side-effects	No statistical differences of opioid-induced AEs	Andreassen et al., 2012 [64]
	Equal side-effects	*CYP2D6* PMs and EMs no statistical differences of incidence of AEs	Andreassen et al., 2012 [64]<br>Slanar et al., 2012 [66]

Table S2. Cytochrome p450 drug interaction. analgesics, antidepressants and anxiolytics involved in *CYP2D6* metabolism.

**Inductors**	**Inhibitors**	**Substrate**
Dexamethasone	Celecoxib	Oxycodone
Oritavancine	Methadone	Tramadol
Rifampicine	Doxepina	Codeine
	Escitalopram	Escitalopram
	Citalopram	Citalopram
	Duloxetina	Atomoxetine
	Paroxetine	Duloxetine
	Sertralina	Paroxetine
	Bupropion	Fluoxetine
	Clomipramina	Venlafaxine
	Clobazam	Doxepine
	Clorpromazine	Nortriptyline
	Haloperidol	Imipramine
	Cocaine	Fluvoxamine
		Amitriptyline
		Clomipramina
		Desipramine
		Metoxi anfetamine
		Clorpromazina
		Lidocaine
		Clonidina
		Haloperidol
		Risperidone
		Dextromethorphan
		Amphetamine
